# Phenotypic and Genotypic Characterization of *Escherichia coli* Causing Urinary Tract Infections in Kidney-Transplanted Patients

**DOI:** 10.3390/jcm8070988

**Published:** 2019-07-07

**Authors:** Jonas Abo Basha, Matthias Kiel, Dennis Görlich, Katharina Schütte-Nütgen, Anika Witten, Hermann Pavenstädt, Barbara C. Kahl, Ulrich Dobrindt, Stefan Reuter

**Affiliations:** 1 Department of Medicine D, Division of General Internal Medicine, Nephrology and Rheumatology, University Hospital Münster, 48149 Münster, Germany; 2 Institute of Hygiene, University of Münster, 48149 Münster, Germany; 3 Institute of Biostatistics and Clinical Research, University of Münster, 48149 Münster, Germany; 4 Institute for Human Genetics, University of Münster, 48149 Münster, Germany; 5 Institute of Medical Microbiology, University Hospital Münster, 48149 Münster, Germany

**Keywords:** Uropathogenic E. coli, UPEC, phylogeny, genomics, antibiotic resistance, virulence traits, kidney transplantation

## Abstract

Urinary tract infection (UTI), frequently caused by uropathogenic Escherichia coli (UPEC), is the most common infection after kidney transplantation (KTx). Untreated, it can lead to urosepsis and impairment of the graft function. We questioned whether the UPEC isolated from KTx patients differed from the UPEC of non-KTx patients. Therefore, we determined the genome sequences of 182 UPEC isolates from KTx and control patients in a large German university clinic and pheno- and genotypically compared these two isolated groups. Resistance to the β-lactams, trimethoprim or trimethoprim/sulfamethoxazole was significantly higher among UPEC from KTx than from control patients, whereas both the isolated groups were highly susceptible to fosfomycin. Accordingly, the gene content conferring resistance to β-lactams or trimethoprim, but also to aminoglycosides, was significantly higher in KTx than in control UPEC isolates. *E. coli* isolates from KTx patients more frequently presented with uncommon UPEC phylogroups expressing higher numbers of plasmid replicons, but interestingly, less UPEC virulence-associated genes than the control group. We conclude that there is no defining subset of virulence traits for UPEC from KTx patients. The clinical history and immunocompromised status of KTx patients enables *E. coli* strains with low uropathogenic potential, but with increased antibiotic resistance to cause UTIs.

## 1. Introduction

Kidney transplantation (KTx) is the best treatment for patients with end-stage renal disease (ESRD). Despite routine screening, treatment and prophylaxis, the prevalence of urinary tract infections (UTIs) in KTx patients is high; more than every third patient is affected [1]. While lower UTI/cystitis usually does not limit graft prognosis, pyelonephritis and urosepsis limit the same, not only in the acute setting, but also in the long run, impairing graft function and prognosis [2,3]. Older age, duration of catheterization, acute rejection periods and deceased donor KTx are known risk factors for UTI after KTx [1]. In addition, female patients are at a higher risk for UTIs. Recently, we concluded that a male donor kidney is a new risk factor [4]. Further potential that predisposes host factors are, e.g., excessive immunosuppression, diabetes mellitus, and the instrumentation of the urinary tract [5]. Uropathogenic *Escherichia coli* (UPEC) are the most common clinical isolates in UTI patients after KTx [6]. UPEC encode virulence factors including adhesins, toxins, capsules, and factors involved in biofilm formation [7]. Resistances to certain antibiotics are common, as patients are frequently treated by antimicrobial compounds, for, e.g., the prophylaxis of *Pneumocystis* pneumonia, UTIs, or in perioperative/periprocedural settings.

As clinical variables and features of patients and UTIs are significantly different from non-transplanted patients, we hypothesized that UPEC isolates from both groups are also different. Therefore, we sought to characterize phylogeny, virulence traits, and antibiotic susceptibility of UPEC from KTx patients and non-transplanted patients to ultimately assist transplant physicians in the management of UTI in KTx patients.

## 2. Experimental Section

Detailed methods are provided in the Supplemental Information.

### 2.1. Study Population

We prospectively collected and analyzed 182 UPEC isolates from 167 patients who were diagnosed with an UPEC-associated UTI between July and December 2016 at the Department of Nephrology or the emergency room at the University Clinic of Muenster. We included 62 KTx patients (71 isolates) and 105 non-transplanted controls (111 isolates). Urine samples were taken in case of clinically relevant UTI symptoms or pyuria and included if ≥10^5^ colony forming units (CFU)/mL urine were detected. Additional blood or respiratory tract samples were collected if required. More than one isolate per patient was included if genotypically different strains were identified from samples.

### 2.2. Patients’ Characteristics

Patients’ and donors’ characteristics were collected from the patients’ files. All non-KTx patients treated at the Department of Nephrology or the emergency room during the study period served as controls. The study was permitted by the local ethics committee (Ethikkommission der Ärztekammer Westfalen-Lippe und der Medizinischen Fakultät der Westfälischen Wilhelms-Universität, No. 2014-381-f-N).

### 2.3. Bacterial Strains and Culture Conditions

We analyzed *E. coli* samples from urine (*n* = 164), blood (*n* = 8), and respiratory tract (*n* = 10). 

### 2.4. Phenotypic Tests

Hemolytic capacity, bacteriocin production, expression of type 1 fimbriae and biofilm formation were analyzed.

### 2.5. Genome Sequencing

Genome sequencing was carried out as described in the Supplemental Information.

### 2.6. Draft Genome Comparison and Typing

Multi-Locus Sequence Typing (MLST) was conducted on all UPEC isolates and UPEC virulence-associated genes were detected.

### 2.7. Statistical Analysis

The data were statistically analyzed using the IBM SPSS statistics 24 for Windows (IBM Corporation, Somers, NY, USA). All baseline variables were described using standard univariate analysis, while bivariate analysis such as the Fisher’s exact test and the t-test were performed for comparing the two groups’ results with each other. The *p*-values were interpreted as exploratory, not confirmatory. If the *p*-values were ≤0.05, the result was considered as statistically significant.

## 3. Results

### 3.1. Patients’ Characteristics

The mean age at the time of infection was comparable between the KTx recipients (79% females) and the control group (70.5% females) (56.7 ± 15.1 vs. 53.45 ± 21.82, respectively). The KTx recipients had a higher BMI (26.19 ± 4.58 kg/m^2^ vs. 24.62 ± 4.41, *p* = 0.0251) and showed a higher frequency of hypertension than control patients (80.6% vs. 35.3%), while the frequency of diabetes did not differ between the groups (Table 1). Causes of ESRD are given in Supplemental Table S1. The recipients’ glomerular filtration rate at the time of UTI was 58.4 ± 32.48 mL/min in KTx patients and 70.21 ± 35.67 mL/min in the control group. To note, the kidney function in KTx patients is based on one working kidney (the transplant) only. More than every fourth recipient and more than every fifth control patient experienced acute renal injury at the time of infection. Lower UTI was observed in ~80% of KTx and controls. 24.5% of KTx and 39.3% controls had been hospitalized for UTI (Table 1). Interestingly, within three months before the UTI diagnosis, KTx patients differed significantly from control patients by being more often hospitalized, having a ureteral stent or urinary catheter, and by antibiotic treatment with cephalosporines, TMP/SMX and fosfomycin mainly for treatments of UTIs and for prophylaxis, e.g., for UTIs and/or Pneumocystis jirovecii infection (Table 1).

### 3.2. Epidemiological Classification of Urine Isolates from KTx and Control Patients

**Figure 1:**
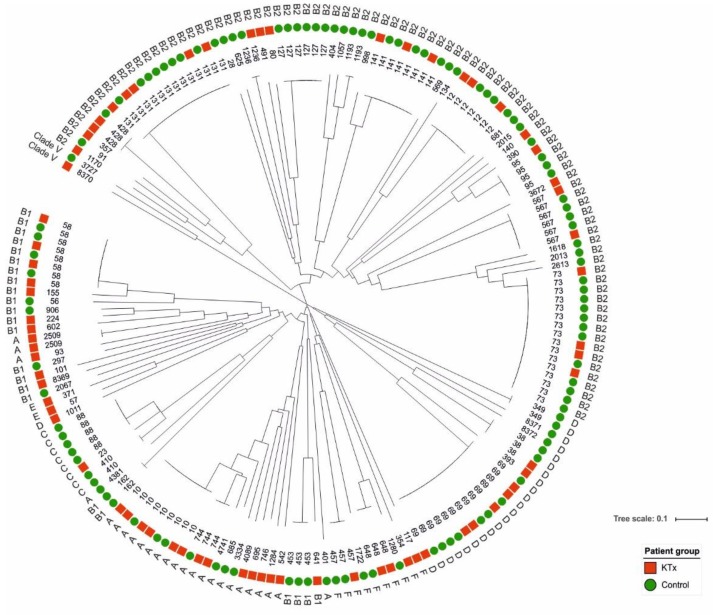
Phylogenetic diversity of the UPEC isolated from KTx and control patients. The neighboring joining tree is based on the MLST of seven housekeeping genes performed with Ridom SeqSphere+ (https://www.ridom.de/seqsphere/) and was created with iTOL (https://itol.embl.de/). The sequence type (ST), the corresponding phylogroup and the isolate group is indicated for each UPEC isolate. The allocation to the two isolate groups (KTx or control) is indicated by different colors. Phylogenetic diversity of UPEC isolated from KTx and control patients. The neighboring joining tree is based on the MLST of seven housekeeping genes and was created with the Ridom SeqSphere+ (https://www.ridom.de/seqsphere/). The sequence type (ST), the corresponding phylogroup and the isolate group (KTx patient yes or no) is indicated for each UPEC isolate. The allocation to different phylogroups is indicated by different colors.

The 182 *E. coli* urine isolates were allocated to phylogenetic lineages and to sequence types (STs) as published by Clermont et al. [8] (Figure 1). 60.5% of all KTx isolates were allocated to phylogroups B2 and A, to which the majority of UPEC primarily belong (Table 2). 

78 different STs were identified. The top ranked STs, representing 93 isolates (51.1% of all isolates), are shown in Supplementary Table S2. Isolates from the KTx and control groups could not be distinguished based on STs. ST10 and ST69 were the most prevalent STs in KTx isolates. ST73 and ST131 were most frequently detected in the control group (Supplementary Table S2). Isolates of KTx patients with a BMI > 25 kg/m^2^ showed a higher number of ST10 and ST131, and a fewer number of ST23 compared to control patients’ isolates. Age did not affect the ST distribution. The prevalence of ST127 and ST73 isolates was markedly higher in the control group supporting our finding that the phylogroup B2 strains are overrepresented in this group.

The O serogroups were highly variable in the urine isolates. Table 3 shows the most frequently predicted O serogroups in 98 isolates (53.8% of all isolates). The O antigen could be typed for 161 isolates leading to the identification of 46 different O antigen types (Table 3). O8 and O89 (9.9% each) were most prevalent in KTx isolates, whereas O6 (15.2%) and O8 (9.8%) were the most prevalent O serotypes in the control group. Typical UPEC O serotypes, i.e., O6, were less prevalent among the KTx isolates. In contrast, KTx isolates more frequently belonged to the O89 serotype than strains of the control group. 

### 3.3. Prevalence of UPEC Virulence-ASSOCIATED genes

**Figure 2:**
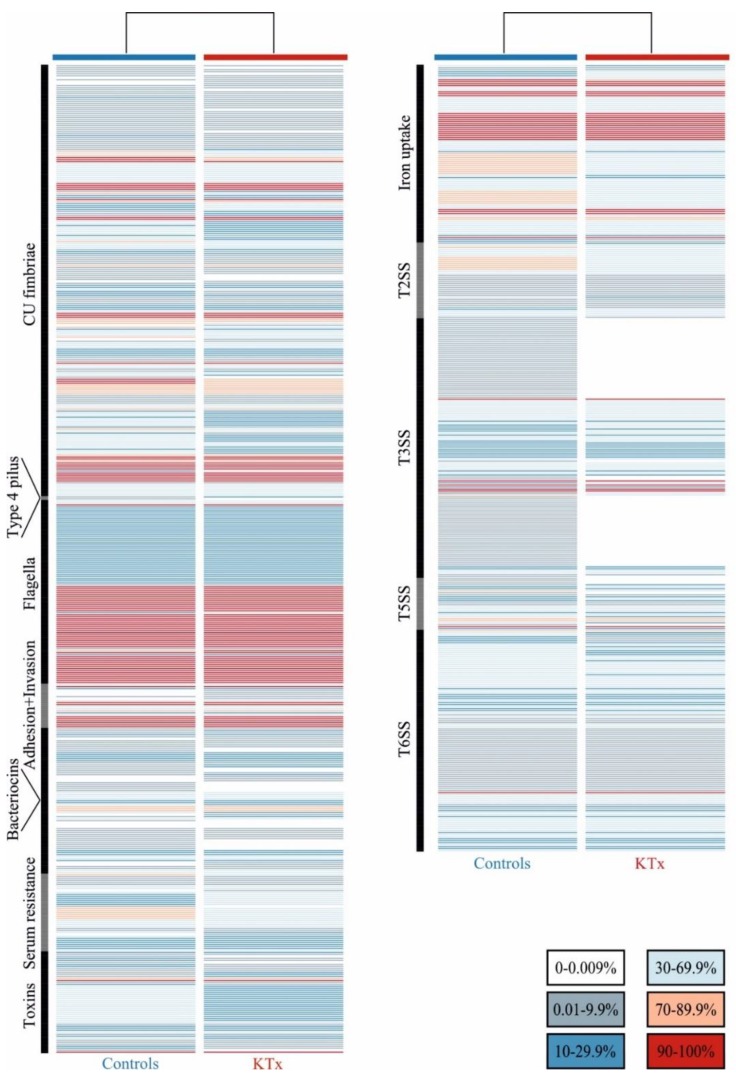
Heatmap displaying the distribution of virulence factors present in KTx and control UPEC isolates. Each row represents one virulence factor. The prevalence of each virulence factor (VF) in KTx and control strains is coded by a color gradient. Vertical black and grey bars indicate the functional VF group: CU-fimbriae = chaperone-usher fimbriae, T2SS = type 2 secretion system, T3SS = type 3 secretion system, T5SS = type 5 secretion system, T6SS = type 2 secretion system. The color code indicates the prevalence of the corresponding VF (calculated as percentage) in the KTx and control group, respectively.

The binary matrix with colored gradation of presence/absence ratios of the virulence factors (VFs) and their functional categories are shown in Figure 2. Of the 1154 VFs tested, 889 were found in at least one isolate. In general, both isolate groups exhibited a similar VF pattern. The overall VF content was slightly higher in the control than in the KTx patient isolates (Figure 2). 113 VFs showed a tendency to be overrepresented either in isolates of the KTx or the control group. Only three VFs (FimB, FocB, SfaB), representing type 1- or S/F1C fimbriae, were significantly associated with control group isolates when a Bonferroni correction was applied (Supplementary Table S3; Supplementary Figure S2). VFs with a tendency of overrepresentation in KTx patient isolates included bacteriocins, adhesins (e.g., Ybg, Yfc, Yhc), ABC transporter (ets), group 4 capsule (GfcABCDE-Etp), type 3 secretion system components, and three autotransporters. VFs enriched in the isolates of control UTI patients comprised VFs which were described for UPEC and phylogroup B2 isolates, including S-/F1C fimbriae, other chaperone-usher family adhesins, group II capsule, the polyketide colibactin and the CjrABC-SenB gene products. The VF binary matrix was used to examine the grouping of isolates according to the VF presence/absence matrix, phylogeny and the isolate group by the principle coordinates analysis. We could not observe a correlation between the source of isolation (KTx vs. control group) and the VF content or phylogroup. Nevertheless, phylogroup B2 strains clustered separately from isolates from other phylogroups.

### 3.4. Antibiotic Susceptibility

A significant number of resistance genes (RGs) was detected in the isolates except for fosfomycin (0.5% of all isolates) and fluoroquinolones (5% of all isolates). UPEC isolates of KTx patients presented more RGs than control UPEC strains (Table 4, Supplementary Table S4). Higher prevalence of the same in KTx patient isolates were observed for trimethoprim, β-lactam, and aminoglycoside resistance determinants. Some resistance genes with high prevalence in the KTx group were less frequently observed in control strains, such as *bla*TEM-1B, *sul*2, *strA* and *strB* (Supplementary Table S4). Clinical susceptibility tests showed that UPEC strains of the KTx and control groups differed significantly in their resistance phenotypes (Table 5).

### 3.5. Plasmid Types

Virulence and resistance determinants are frequently located on plasmids. We identified 25 different plasmid replicon types in 149 isolates. UPEC isolates of the control group were more often plasmid-free than isolates from the KTx group. IncFIB, IncFII, Col156 and Inc1 were the most frequently identified replicon types among all strains. IncFIB, IncFII, IncFIC, IncI1 and IncQ1 replicons were more prevalent in the KTx than in the control group. The Col156 replicon was more frequently found in control isolates. The seven most prevalent plasmid types are summarized in Table 6.

### 3.6. Phenotypic Assays

**Figure 3:**
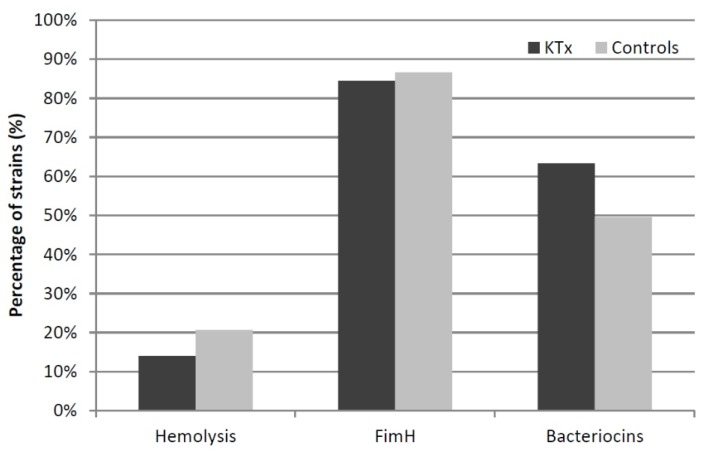
Phenotypic characteristics of the KTx and control UPEC isolates. The percentage of strains phenotypically tested positive for the expression of α-hemolysin, type 1 fimbriae and bacteriocins killing *E.coli* DH5α has been compared between the KTx and control groups.

**Figure 4:**
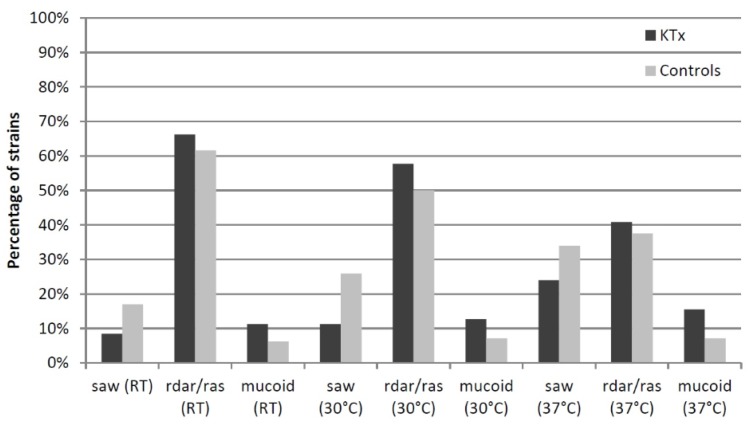
Expression of biofilm morphotypes in KTx and control UPEC isolates. The percentage of strains expressing different biofilm morphotypes at different growth temperatures is compared between the KTx and control groups.

Almost all the analyzed UPEC isolates (97.8%) carried the type 1 fimbrial adhesin gene *fimH*. 156 strains (85.7%), and functionally expressed type 1 fimbriae (KTx: 84.5% vs. controls: 86.5%). α-Hemolysin was produced in 18.1% of isolates without any significant differences between both groups. UPEC from KTx patients (63.4%) more often expressed often bacteriocins killing *E. coli* DH5α than control UPEC (49.5%) (Figure 3). Cellulose and curli expression are important for biofilm formation of *E. coli* (Figure 4, Supplementary Tables S5 and S6). The corresponding rdar/ras morphotype expression was more prevalent at lower growth temperatures and decreased with increasing temperature. Rdar/ras morphotype expression was more frequently observed in the UPEC of KTx patients. Increased expression of the “saw” morphotype correlated with increasing incubation temperature and occurred more often in the control group. The rare mucoid morphotype appeared more often in the KTx isolates.

## 4. Discussion

UTI results from complex pathogen–host interactions and is affected by host risk factors, bacterial traits and the host immune response [9]. Risk factors for uncomplicated and complicated UTI in adults have been defined before [10]. Genetic factors involved in host susceptibility to acute pyelonephritis have been described including polymorphisms that reduce expression of the interferon regulatory factor 3 (IRF3) or CXCR1 coding for the IL-8 receptor [11].

Our main objectives were to evaluate if the KTx-related factors, such as antibiotic therapy, immunosuppression or host characteristics, impact on UPEC, the major pathogen causing UTI after KTx [1]. We herein present the genotypic and phenotypic characteristics of the UPEC isolated from KTx patients first.

Prior use of antibiotics promotes the risk of UTI due to (multi-)resistant uropathogens as frequent antibiotic prescription leads to a selection of more resistant bacteria, which is also highlighted by our data which shows increased antibiotic resistances in the KTx group isolates (Table 4 and Table 5). Antibiotic susceptibility rates are significantly lower in hospital- than in community-acquired UTIs [12]. Immunocompromised patients often display co-morbidities and are more often hospitalized than immunocompetent patients. Accordingly, the former are more likely to develop UTI caused by multi-drug resistant pathogens [13]. Accordingly, comparing the KTx patients with controls three months prior to the UTI diagnosis, there were significant differences in clinical parameters such as hospitalization rate or application of devices (ureteral stents, urinary catheter); all of which seem to facilitate UTIs in KTx patients. In the transplant setting, invasive procedures such as the instrumentation of the urinary tract as well as antimicrobial prophylaxis against opportunistic microorganisms and treatment of infections are common and are more frequently performed than in controls (Table 1). These interventions affect the microbiome composition, fostering urinary dysbiosis in KTx patients [14]. *E. coli* was found seven times more often in KTx recipients than in controls. Rani and colleagues concluded that UTIs follow the disruption of the local microbial homeostasis and arise from bacteria being part of the colonizing microbiome [14]. Patients suffering from recurrent UTI or with frequent occurrence of symptomatic episodes are at a higher risk of being infected with resistant uropathogenic strains [15,16,17,18]. Interestingly, we more frequently observed treated UTIs in KTx patients within a three-month period before the sample acquisition. Thus, factors promoting the selection of virulent UPEC should be minimized and therapeutic or probiotic modification of the colonizing microbiome may mitigate frequency and/or severity of UTIs. Against this background, depletion of intestinal reservoirs of UPEC [19] or fecal microbiota transplantation [20] are promising approaches to tackling such infections.

In particular, treatment of asymptomatic bacteriuria led to a significant exposure to antibiotics in KTx patients. Recent studies suggest against the treatment this condition, which seems to be a step in the right direction [21].

In Germany, the resistance rate of UPEC in uncomplicated UTI was 25.9% for TMP/SMX, 34.9% for ampicillin, 3.7% for ciprofloxacin, and 0.8% for fosfomycin [22]. International data shows higher resistance rates for all antibiotic classes in UPEC from KTx recipients [6,23,24]. Similarly, we found relevant resistance rates among our KTx patients as they have received more frequently antibiotic treatments, e.g., with TMP/SMX, and the UPEC carried more trimethoprim, sulfonamide, and β-lactam resistance genes. In particular, the carriage of blaTEM-1B, strA and strB genes was significantly higher than in control strains (Table 4, Supplementary Table S4). Accordingly, the high resistance rates to antibiotics are clinically important (Table 5). Especially for TMP/SMX, one should balance the high resistance rate against the benefit of the *P. jirovecii* prophylaxis [25]. In line with international findings, we only identified a few fosfomycin resistance determinants leading to reasonable susceptibility rates of UPEC [26]. In this regard, our data are relevant for the clinical decision-making.

Several reports focused on “common” O serogroups linked with UTIs [27], but data regarding O serogroups of *E. coli* causing UTIs in KTx patients is limited. In an analysis of 40 urine isolates from KTx patients, *E. coli* ST131 (O25:H4) was identified to be the most prevalent clone [28]. We detected most of the previously reported “common” UPEC O serogroups, including O25 in KTx isolates with the highest prevalence of serogroups O8 and O89 [28]. In general, distinct O serogroups could not be correlated with KTx isolates (Table 3). In contrast to Rice et al., who proposed a unique pattern of UPEC O serogroups in their US patient cohort with acute kidney injury (AKI) at the time of UTI, we did not find such an association [28]. Our AKI rate was lower and there was a similarity between KTx and controls. The fact that the renal function of KTx patients is based on one working kidney (the transplant) only might confound this analysis because two kidneys might better compensate for affections, especially when one kidney is infected (pyelonephritis). Also, the definition of AKI varied between studies. Interestingly, the hospitalization rate was lower in KTx patients than in controls, although the types of UTI were comparable between the groups.

The majority of *E. coli* strains with an increased potential to cause UTIs belong to phylogroups B2 and D, whereas isolates of other phylogenetic lineages display a reduced extraintestinal virulence potential. Phylogroups A and B1 often comprise commensals or diarrheagenic pathogens [29]. In our study, the B2 strains were overrepresented among UPEC isolates of the control group, while phylogroup A strains occurred more frequently in KTx patients. Similarly, Tashk et al. observed that *E. coli* urine isolates from KTx patients more frequently belonged to phylogroups which are uncommon for UPEC, such as phylogroups A, B1, and F, while typical UPEC phylogroups (B2 and D) were overrepresented among the fecal strains of these patients [30].

Recent analyses of UPEC phylogeny describe ST14, ST69, ST73, ST95 and ST131 as the most prevalent clones causing UTI [31]. ST131 is a pandemic clone responsible for a high incidence of extraintestinal infections worldwide [32]. Among our isolates, ST131 was one of the three most prevalent STs. In congruence with the results obtained for the phylogroup distribution, seven out of the ten most prevalent STs in our cohort represent phylogenetic lineages B2 and D (Supplementary Table S1), whereas ST127 significantly correlated with UPEC isolates of controls, and no ST was significantly associated with KTx UPEC.

Only a few selected UPEC virulence traits have been studied in the isolates of KTx patients (*n* = 36) and of non-transplanted patients (*n* = 27) without differences between groups [33]. This result was confirmed in our genome-wide approach. Although 113 virulence-associated gene products showed the tendency to be overrepresented in either the KTx or the control group, only three VFs (FimB, SfaB, FocB) proved to be significantly associated with one group, i.e., the control group. S/F1C fimbriae, as well as the other VFs, are important virulence-associated factors of *E. coli* strains of the phylogroups B2 and D [34]. Additionally, the presence of the B2 phylogroup-enriched cjrABC-senB gene products correlates with increased urovirulence [35,36]. Our PCoA underlines that the prevalence of VFs does not strictly correlate with the isolate groups, but rather with their phylogenetic background (Supplementary Figure S3). As the phylogenetic background plays a critical role for the overall genome content, the VF distribution correlates with phylogeny [37,38]. Typical phylogroup B2 VFs were more frequently found in controls; while in the KTx group, other VFs, including phylogroup A-associated Yhc fimbriae, were overrepresented.

Our analysis of plasmid replicons present in both isolate groups partly corresponds with recent studies [39]. Plasmids are common in UPEC with IncFIB, with IncFII being the most common replicon types. This is in congruence with our data. We identified a large number of colicin plasmids. Col-type plasmids frequently harbor resistance, as well as UPEC virulence-associated genes. Interestingly, isolates from control groups carried almost twice as many colicin plasmids as isolates from KTx patients.

Phenotypic characteristics like α-hemolysin, bacteriocin, and type 1 fimbriae expression or biofilm formation have not been studied in UPEC from KTx patients so far. In this isolate group, the bacteriocin expression was significantly higher than in the UPEC of controls. The rdar/ras morphotype was the most prevalent biofilm-related property in both isolate groups, always slightly higher when expressed in KTx patients’ isolates. The slightly increased ability of the KTx isolates to express the rdar biofilm morphotype is an aspect that needs further investigations.

In summary, we show significant differences regarding the phylogenetic background of the UPEC isolates from KTx patients compared to non-transplanted patients. As the prevalence of individual VFs correlates with the phylogroup allocation, the observed differences in the VF distribution among the two isolate groups can be explained. Regarding their phylogenetic background, plasmid content and overall VF distribution, *E. coli* isolates from UTI in non-KTx patients who often share these traits with the typical UPEC described for uncomplicated UTI cases. In contrast, isolates from KTx patients included typical UPEC clones and carried less UPEC virulence-associated genes at a lesser frequency. These strains rather exhibited a weak uropathogenic potential. Our findings suggest that in immunocompromised KTx patients, even *E. coli* strains lacking typical UPEC VFs can cause UTIs, whereas the establishment of UTI in non-transplanted patients requires an increased uropathogenic potential. Clinically important are our findings that UPEC from KTx patients show a higher resistance to several groups of commonly used antibiotics, phenotypically and genotypically. Our study adds important aspects to the concept that UTI establishment results from multiple independent factors including the pathogenic potential of the invading pathogens, its interaction with the host and the host’s clinical history and susceptibility.

## Figures and Tables

**Table 1:** Clinical data.

**Clinical data at UTI diagnosis**			
	**KTx Recipients**	**Controls**	***p***
Sex (female/male)	49 (79%)/13 (21%)	74 (70.5%)/31 (29.5%)	0.1512
Age (mean/median/σ in yr)	56.67/58.75/15.09	53.45/ 55/21.82	0.2902
BMI (mean/median/σ)	26.19/26.03/4.58	24.62/24.21/4.41	**0.0251**
Hypertension (%)	80.6%	35.3%	**0**
Diabetes mellitus (%)	21%	14.7%	0.3922
Immunosuppression (%)	100%	9.7%	**0**
Previous tumor diagnosis (%)	0%	10.7%	**0.0072**
Time from last KTx to UTI (mean/median/σ in yr)	5.4/3.2/6.2	-	-
Ureteral stent or urinary catheter (%)	16.1%	5.8%	0.0539
Lower UTI (%)	80.3%	80.4%	1
Upper UTI (%)	19.7%	19.6%	1
eGFR (mean/median/σ in mL/min)	58.4/54.45/32.48	70.21/66.5/35.67	**0.0406**
Acute kidney injury (%)	28.3%	23.1%	0.5658
Hospitalization	24.5%	39.3%	0.0527
**Clinical data of a three-month period before the UTI diagnosis**			
Hospitalization	40.3%	20%	0.0068
Surgery	21%	10.5%	0.0709
Ureteral stent or urinary catheter	29%	12.4%	0.0125
**Antibiotic therapy**			
Beta-lactam	6.5%	1.9%	0.1961
Cephalosporine	25.8%	4.8%	0.0002
Fluoroquinolone	9.7%	2.9%	0.0787
Trimethoprim/ Sulfonamide	30.6%	2.9%	0
Fosfomycin	11.3%	2.9%	0.0401
Carbapenem	0%	1%	1
**Reason for antibiotic treatment**			
Prophylaxis	29%	4.8%	0
UTI	25.8%	7.6%	0.0024
Other infection	12.9%	7.6%	0.2855

σ = standard variation, BMI= body mass index, eGFR = estimated glomerular filtration rate, KTx = kidney transplantation, *p*-values were obtained using t-test.

**Table 2:** Distribution of phylogroups in relation to KTx and control UPEC isolates.

Phylogroup	Total	KTx		Controls		*p*
	No. of Isolates	No. of Isolates	%	No. of Isolates	%	
A	25	16	22.5	9	8.1	**0.0061**
B1	24	10	14.1	14	12.6	0.4706
B2	88	27	38	61	55	**0.0394**
C	8	1	1.4	7	6.3	0.1117
D	23	9	12.7	14	12.6	0.5805
E	2	2	2.8	0	0	0.1509
F	10	5	7	5	4.5	0.3389
Clade V	2	1	1.4	1	0.9	0.6293

No. = number, KTx = strains of kidney transplanted patients, *p*-values were obtained using Fisher’s exact test.

**Table 3:** Distribution of O serotypes in relation to KTx and control UPEC isolates.

Serogroups	Total	KTx		Controls		*p*
	No. of Isolates	No. of Isolates	%	No. of Isolates	%	
O2	14	5	7	9	7.1	0.5159
O4	8	3	4.2	5	5	0.6187
O6	20	3	4.2	17	15.2	**0.0148**
O8	18	7	9.9	11	9.8	0.6013
O15	10	6	8.5	4	3.6	0.1435
O16	5	2	2.8	3	2.7	0.6485
O25	11	4	5.6	7	6.3	0.5608
O83	5	1	1.4	4	3.6	0.3515
O89	10	7	9.9	3	2.7	**0.0432**
not typeable	21	10	14.1	11	10	nd
other serotypes	60	23	32.4	31	33.9	nd

No. = Number, KTx = strains of kidney transplanted patients, nd = not determined, *p*-values were obtained using Fisher’s exact test.

**Table 4:** Distribution of resistance genes (RGs) present in relation to KTx and control UPEC isolates.

Antibiotic		>1 RGs present		No RGs present		*p*
		No. of Isolates	%	No. of Isolates	%	
Beta-Lactam	KTx	42	59.2	29	40.8	**0.0033**
	Controls	41	36.9	70	63.1	
Trimethoprim	KTx	34	47.9	37	52.1	**0.0061**
	Controls	31	27.9	80	72.1	
Sulfonamides	KTx	35	49.3	36	50.7	0.0763
	Controls	40	36.0	71	64.0	
Fosfomycin	KTx	0	0.0	71	100	1
	Controls	1	0.9	110	99.1	
Fluoroquinolones	KTx	3	4.2	68	95.8	1
	Controls	6	5.4	105	94.6	
Aminoglycosides	KTx	42	59.2	29	40.8	**0.0101**
	Controls	44	39.6	67	60.4	

No. = number, KTx = strains of kidney transplanted patients, RG = resistance gene, nd = not determined, *p*-values were obtained using Fisher’s exact test.

**Table 5:** Susceptibility patterns of KTx and control UPEC isolates.

Antibiotic		Susceptible		Intermediate		Resistant		*p*
		No. of Isolates	%	No. of Isolates	%	No. of Isolates	%	
Beta- Lactam (AMP+AMX)	KTx	27	38	0	0	44	62	**0.0015**
	Controls	69	62.2	0	0	42	37.8	
Beta- Lactam + inhibitor (SAM)	KTx	50	70.4	0	0	21	29.6	0.1734
	Controls	88	79.3	0	0	23	20.7	
Beta- Lactam (CFX)	KTx	58	81.7	0	0	13	18.3	0.2147
	Controls	98	88.3	0	0	13	11.7	
Trimethoprim	KTx	33	50	0	0	33	50	**0.0029**
	Controls	70	72.9	0	0	26	27.1	
TMP/SMX	KTx	37	52.1	0	0	34	47.9	**0.0132**
	Controls	78	70.3	0	0	33	29.7	
Fosfomycin	KTx	66	98.5	0	0	1	1.5	1
	Controls	96	98	0	0	2	2	
Fluoroqinolones (CIP+LVX)	KTx	55	77.5	1	1.4	15	21.1	0.6588
	Controls	92	82.9	1	0.9	18	16.2	
Aminoglycosides (GEN)	KTx	66	93	0	0	5	7	0.4637
	Controls	106	95.5	0	0	5	4.5	

No. = number, KTx = strains of kidney transplanted patients, AMP = ampicillin, AMX = amoxicillin, SAM= ampicillin/sulbactam, CFX = Cefuroxime, TMP = trimethoprim, SMX = sulfamethoxazole, CIP = ciprofloxacin, LVX = levofloxacin, GEN = gentamicin, *p*-values were obtained using Fisher’s exact test.

**Table 6:** Distribution of the most frequent plasmid replicon types in relation to KTx and control UPEC isolates.

Plasmid Type	Total No. of Isolates	KTx		Controls		*p*
		No. of Isolates	%	No. of Isolates	%	
IncFIA	24	10	14.1	14	12.6	0.4706
IncFIB	114	49	69	65	58.6	0.1025
IncFIC	27	16	22.5	11	9.9	**0.0178**
IncFII	100	40	56.3	60	54.1	0.4411
IncI1	33	17	23.9	16	14.4	0.0773
IncQ1	20	13	18.3	7	6.3	**0.0120**
Col156	35	9	12.7	26	23.4	0.0526
Other replicons	68	30	42.3	38	34.2	0.1751
None	33	7	9.9	26	23.4	**0.0150**

No. = number, KTx = strains of kidney transplanted patients, *p*-values were obtained using Fisher’s exact test.

## Supplementary Materials

The following are available online at https://www.mdpi.com/2077-0383/8/7/988/s1. Figure S1: Representative biofilm morphotypes expressed by UPEC isolates on Congo Red agar plates, Figure S2: Manhattan Plot of Fisher’s exact test *p*-values for the prevalence of individual virulence factors (VF) in an isolate group (KTx or control). The red line separates VFs with *p*-values < 0.05 from VFs without significant association with an isolate group. The green line separates VFs with significant association (*p* < 0.05) after the Bonferroni correction, Figure S3: Principal Coordinates Analysis (PCoA) to examine the grouping of UPEC isolates according to the presence/absence of virulence-associated genes, their phylogenetic group and their isolate group (KTx vs. control group). The axes are scaled with eigenvalue scaling using the square root of the eigenvalue and indicate the percentage of variation explained in the PCoA. KTx strains are marked with (+) and control strains are marked with (•). The phylogroup of each strain is color coded (phylogroup A = blue, phylogroup B1 = light green, phylogroup B2 = dark green, phylogroup C = brown, phylogroup D = pink, phylogroup E = red, phylogroup F = orange, clade V = black), Table S1: Additional KTx recipient and donor data, Table S2: Distribution of sequence types (STs) in relation to KTx and control UPEC isolates, Table S3: List of VFs significantly associated (Fisher’s exact test) with either KTx or control UPEC isolates, Table S4: Resistance genes (RGs) with the largest differences between KTx and control isolates, Table S1: Distribution of the rdar/ras, saw and mucoid morphotypes expressed by KTx and control UPEC isolates, Table S6: Calcofluor binding KTx and control UPEC isolates.
