# Supplementary material for: Phenotypic and Genotypic Characterization of Escherichia coli Causing Urinary Tract Infections in Kidney-Transplanted Patients
Source: J Clin Med. 2019 Jul 7;8(7):988. doi: 10.3390/jcm8070988 (PMC6678207; doi:10.3390/jcm8070988)
Supplement: Supplementary file 1 [file jcm-08-00988-s001.pdf]

Journal of Clinical Medicine

MDPI

Supplementary Material

Materials and Methods

Patients' characteristics

Patients' characteristics (age, sex, body mass index, time between KTx and UTI, underlying renal diseases, number of pre-transplantations, European Senior Program (ESP), type of immunosuppression, hypertension, diabetes, and malignancy) and donor characteristics (sex and age) were collected from the patient`s file (Table 1). Occurrence of acute renal injury (AKI) was assessed based on the AKIN classification (1) with baseline serum creatinine as the average of 1–3 measures in the previous 2–12 weeks prior to the UTI. As we had not sufficient data on urinary output we used creatinine-based definition of AKI only. The eGFR on the day of UTI was calculated using the CKD-EPI (Chronic Kidney Disease Epidemiology Collaboration) formula.

Data of all patients were anonymized prior to analysis. Induction therapy was performed with anti-interleukin-2 receptor antibodies (86.7%), anti-human T-lymphocyte immunoglobulin (10%), or alemtuzumab (1.7%) leaving one patient without induction therapy. Immunosuppressive regimen consisted of tacrolimus, mycophenolate mofetil or mycophenolic acid with or without prednisolone in all patients except five patients who received everolimus and five patients who were treated with belatacept. The antibiotic standard regimen in KTx patients consists of a perioperative single shot antibiosis with cefuroxime (at transplantation). Thereafter, a Pneumocystis jirovecii prophylaxis is given for three months using trimethoprim-sulfamethoxazole. An additional prophylaxis for Pneumocystis jirovecii is applied for three months in patients with treated allograft rejection. Samples were taken on average 5.4 ± 6.2 years after KTx. As controls served all non-KTx patients treated at the Department of Nephrology or the emergency room during the study period.

Bacterial strains and culture conditions

We analyzed *E. coli* samples from urine (n = 164), blood (n = 8), and respiratory tract (n = 10). Taxonomic assignment and *E. coli* species allocation of the clinical isolates were performed by MALDI-TOF analysis (BrukerDaltonics, Bremen, Germany). For antibiotic susceptibility testing, the VITEK2 system (bioMérieux, Nürtingen, Germany) was used. UPEC were stored in lysogeny broth (LB) containing 25 % glycerol at −80 °C. Before further testing, UPEC were grown on LB agar overnight at 37 °C.

Phenotypic Tests

Hemolytic capacity. The bacterial isolates were streaked out on Columbia blood agar containing 5% sheep blood (Oxoid, Wesel, Germany) and incubated overnight at 37 °C. The result was positive, if a distinct hemolytic zone was visible around the colonies.

Bacteriocin production. Bacteriocin expression was tested by a modified soft agar overlay assay (Fredericq, 1957). The isolates were inoculated on M9 soft agar (0.75% agar) seeded with indicator strain DH5α. Growth of the inoculated clinical isolates and secretion of antagonistic factors into the agar interferes with growth of indicator strain DH5α. The test was carried out at 37 °C overnight and regarded as positive when a clear halo appeared around the cultures due to growth inhibition or killing of the indicator strain DH5α.

Type 1 fimbriae expression. The expression of D-mannose binding type 1 fimbriae was quantified by agglutination of *Saccharomyces cerevisiae* cells. Aliquots of bacterial overnight cultures in LB were incubated with a yeast suspension (10 mg/mL dry weight) and agglutination susceptible to inhibition by D-mannose (3 %) was scored visually based on the aggregation and precipitation of the cells. The result was considered as positive if the bacteria aggregated with yeast in the absence of mannose.

Biofilm formation. To characterize biofilm formation, each strain was analyzed on Congo Red (CR) and Calcofluor White (CF) agar plates. Expression of the 'red dry and rough' (rdar) multicellular behaviour was visualized by cultivation on LB without salt agar supplemented with 40 µg/mL Congo Red (Sigma-Aldrich, Deisenhofen, Germany) and 20 µg/mL Coomassie Brilliant Blue G-250 (Sigma-Aldrich, Deisenhofen, Germany) (2). Cellulose expression was assayed on LB without salt agar supplemented with 160 µg/mL Calcofluor White (Sigma-Aldrich, Deisenhofen, Germany). Expression of cellulose was visualized by fluorescence under UV light (366 nm). The strains were grown at room temperature (RT), 30 °C and 37 °C for 72 h. On CR plates *E. coli* expresses different morphotypes (Supplementary Figure 1).

Genome sequencing

The DNA of all isolates was extracted by using MagAttract HMW DNA Kit (Qiagen, Hilden, Germany). The extracted high molecular weight genomic DNA was quantified and assessed for purity using the NanoDrop 2000c spectrophotometer (ThermoFischer Scientific, Darmstadt, Germany) and agarose gel electrophoresis. To prepare 500 bp paired end libraries of all UPEC isolates we used the Nextera XT DNA Library Preparation kit (Illumina, Eindhoven, The Netherlands). Libraries were sequenced on the Illumina NextSeq 500 sequencing platform using v2 (300 cycles) sequencing chemistry.

Assembly

Fast software (v0.11.5) was used to control the quality of the received raw reads and they were assembled using SPAdes v3.5 (3). Contigs smaller than 1000 bp were excluded and quality control of the assemblies was performed using QUAST v2.3 (4). *De novo* assembled genomes were analyzed if the number of contigs was <1000, N50 values were >5000 bp and L50 values were <50. The genome sequences had on average 83.3 contigs, N50 values of 233,542.1 bp and L50 values of 8.8.

Draft genome comparison and typing

Multi-Locus Sequence Typing (MLST) was conducted on all UPEC isolates using the ClermonTyper software (https://github.com/A-BN/ClermonTyping). Visualization of the phylogenetic relatedness of the isolates based on the MLST results was performed with the SeqSphere software (Ridom GmbH, Münster, Germany, http://www.ridom.de/seqsphere/). For further in silico analysis a web-based tool (https://cge.cbs.dtu.dk/services/) was used to identify serotypes (5), plasmid types (6) and resistance genes (RGs) for antibiotics (ABs) such as fosfomycin, fluoroquinolones, trimethoprim, beta-lactam ABs, sulfonamides and aminoglycosides (7).

Detection of virulence-associated factors encoded by the different UPEC isolates

The tools Prokka (v1.12) (8) and Proteinortho (v5.15) (9) were used to determine coding sequences (CDS), annotate the genomes and to detect orthologous genes. An in-house database was applied to determine virulence factors (VFs) encoded in the UPEC genomes. The *E. coli* VF collection (v0.1) (https://github.com/aleimba/ecoli_VF_collection) was extended and finally included a total of 12 VF groups containing 1154 deduced protein sequences of virulence-associated genes. Absent or present VFs were processed with the ‘prot_finder’ package of the ‘bac-genomics-scripts’ collection (https://github.com/aleimba/bac-genomics-scripts). To identify VFs, which are significantly associated ($p < 0.05$ and with a Bonferroni correction) with either the KTx or control group, we used Fisher’s Exact test provided in R (v3.2.5). The Fisher’s exact test p values are visualized as a Manhattan plot with R package ggplot2 (v2.2.0) (10) (Supplementary Figure 2). The heatmap of the corresponding matrix was generated with R (v3.2.5) (11) with a color gradient representing the ratio of present/absent VFs within KTx patients and controls.

The relationship of KTx and control UPEC isolates with the prevalence of virulence factors and phylogroups was examined via multivariate analyses. A principle coordinates analysis (PCoA) was plotted based on a Bray-Curtis similarity matrix of the BLASTP hits with PAST (v3.18) and a transformation exponent of c = 2 (12, 13). PCoA allows to maximally correlate the distances in the ordination diagram with the linear

distance measures in the distance matrix. Here, the PCoA was used to examine the grouping of UPEC strains according to the virulence- and fitness gene matrix and their isolation from KTx or control patients (14, 15).

Statistical analysis

The data were statistically analyzed using IBM SPSS statistics 24 for Windows (IBM Corporation, Somers, NY, USA). Baseline variables were described using standard univariate analysis. To compare both groups Fishers' exact test and the t test were used. Patients' characteristics were tested and compared by the t test while Fisher's exact test was used for bivariate analysis of differences in phenotype and genotype between both groups. P values were interpreted as exploratory, not confirmatory. If *p* values were ≤0.05 the result was considered as statistically significant.

References:

1. Mehta RL, Kellum JA, Shah SV, Molitoris BA, Ronco C, Warnock DG, et al. Acute Kidney Injury Network: report of an initiative to improve outcomes in acute kidney injury. Crit Care. 2007;11(2):R31.
2. Romling U, Sierralta WD, Eriksson K, Normark S. Multicellular and aggregative behaviour of Salmonella typhimurium strains is controlled by mutations in the agfD promoter. Mol Microbiol. 1998 Apr;28(2):249-64.
3. Bankevich A, Nurk S, Antipov D, Gurevich AA, Dvorkin M, Kulikov AS, et al. SPAdes: a new genome assembly algorithm and its applications to single-cell sequencing. J Comput Biol. 2012 May;19(5):455-77.
4. Gurevich A, Saveliev V, Vyahhi N, Tesler G. QUAST: quality assessment tool for genome assemblies. Bioinformatics. 2013 Apr 15;29(8):1072-5.
5. Joensen KG, Tetzschner AM, Iguchi A, Aarestrup FM, Scheutz F. Rapid and Easy In Silico Serotyping of Escherichia coli Isolates by Use of Whole-Genome Sequencing Data. J Clin Microbiol. 2015 Aug;53(8):2410-26.
6. Carattoli A, Zankari E, Garcia-Fernandez A, Voldby Larsen M, Lund O, Villa L, et al. In silico detection and typing of plasmids using PlasmidFinder and plasmid multilocus sequence typing. Antimicrob Agents Chemother. 2014 Jul;58(7):3895-903.
7. Zankari E, Hasman H, Cosentino S, Vestergaard M, Rasmussen S, Lund O, et al. Identification of acquired antimicrobial resistance genes. J Antimicrob Chemother. 2012 Nov;67(11):2640-4.
8. Seemann T. Prokka: rapid prokaryotic genome annotation. Bioinformatics. 2014 Jul 15;30(14):2068-9.
9. Lechner M, Findeiss S, Steiner L, Marz M, Stadler PF, Prohaska SJ. Proteinortho: detection of (co-)orthologs in large-scale analysis. BMC Bioinformatics. 2011 Apr 28;12:124,2105-12-124.
10. Wickham H, editor. ggplot2 - Elegant Graphics for Data Analysis. 1st ed. Springer New York; 2009.
11. R: A language and environment for statistical computing. [Internet].; 2016 [updated 2016-04-14;]. Available from: https://www.r-project.org/.
12. Hammer O, Harper DAT, Ryan PD. PAST: paleontological statistics soft-ware package for education and data analysis. Palaeontologia Electronica [Internet]. 2001 22.06.2001. Available from: https://palaeo-electronica.org/2001_1/past/past.pdf.
13. Legendre P, Legendre L, editors. Numerical Ecology. 24 2nd Edition ed. Amsterdam, Netherlands: Elsevier Science; 1998.
14. Quinn GP, Keough MJ, editors. Experimental Design and Data Analysis for Biologists. 1st ed. Cambridge: Cambridge University Press; 2002.
15. Ramette A. Multivariate analyses in microbial ecology. FEMS Microbiol Ecol. 2007 Nov;62(2):142-60.

Supplementary Table 1. Additional KTx recipient and donor data.

**Recipient data**	
**Number of previous KTx (>1 in %)**	11.3
Age at KTx (mean/median/σ in years)	52.00 / 53.18 / 14.91
Induction therapy (None / IL-2 receptor antagonist/ Alemtuzumab / rabbit-derived antithymocyte globulin in %)	1.7 / 86.7 / 1.7 / 10.0
European Senior Program * (%)	17.7
Diagnosis of ESRD	
Glomerulonphritis	23 (37.1 %)
Autosomal dominant PKD	10 (16.1 %)
Reflux nephropathy	6 (9.7 %)
Diabetic nephropathy	3 (4.8 %)
Single kidney	2 (3.2 %)
Aristolochic acid nephropathy	2 (3.2 %)
Hypertensive nephrosclerosis	2 (3.2 %)
Amyloidosis	2 (3.2 %)
Other	12 (19.4%)
**Donor data**	
Gender (female/male)	37 (61.7%) / 23 (38.3%)
Age (mean/median/σ in years)	53.12 / 55.00 / 13.88

σ = standard variation, KTx= kidney transplantation, PKD= polycystic kidney disease * patients transplanted within the Eurotransplant European Senior Program.

Supplementary Table 2. Distribution of sequence types (STs) in relation to KTx and control UPEC isolates.

Sequence Type	Total	KTx		Controls		*p*
	No. of isolates	No. of isolates	%	No. of isolates	%	
ST10	9	6	8.5	3	2.7	0.0833
ST12	7	3	4.2	4	3.6	0.5605
ST58	8	4	6.6	4	3.6	0.3813
ST69	14	6	8.5	8	7.1	0.4840
ST73	16	4	5.6	12	10.7	0.1758
ST88	5	0	0.0	5	5.0	0.0814
ST127	6	0	0.0	6	5.4	**0.0488**
ST131	14	5	7.0	9	8.0	0.5159
ST141	8	3	4.2	5	5.0	0.6187
ST567	6	2	2.8	4	3.6	0.5651
other STs	89	38	53.5	51	45.9	0.1990

No.= number, KTx= strains of kidney transplanted patients, p values were obtained using Fisher's exact test.

Supplementary Table 3. List of VFs significantly associated (Fisher's exact test) with either KTx or control UPEC isolates.

Virulence factor	Functional category	*p* Value	overrepresented in KTx group	overrepresented in Control group
Cbi	Bacteriocins	1,49E+04	+	
CblA	Bacteriocins	2,71E+04	+	
Cma	Bacteriocins	4,40E+03	+	
Cmi	Bacteriocins	1,49E+04	+	
Cna	Bacteriocins	4,40E+03	+	
EcSMS35_B0	Bacteriocins	7,89E+03	+	

007				
McbC	Bacteriocins	6,01E+03	+	
Cba	Bacteriocins	2,71E+04	+	
Hra1	Adhesion_invasi on	2,34E+04	+	
YbgD	CU_fimbriae	3,42E+04	+	
YbgQ	CU_fimbriae	3,31E+04	+	
YfcP	CU_fimbriae	2,79E+04	+	
YfcR	CU_fimbriae	1,12E+04	+	
YfcS	CU_fimbriae	4,46E+04	+	
YhcA	CU_fimbriae	7,79E+03	+	
YhcD	CU_fimbriae	7,79E+03	+	
YhcF	CU_fimbriae	7,79E+03	+	
FanC	CU_fimbriae	3,36E+04	+	
AufA	CU_fimbriae	4,03E+04		+
AufD	CU_fimbriae	2,70E+04		+
CssD	CU_fimbriae	3,03E+03		+
Dra2E	CU_fimbriae	3,36E+04		+
EC042_1639	CU_fimbriae	4,15E+04		+
FaeD	CU_fimbriae	1,95E+04		+
**FimB**	**CU_fimbriae**	**1,97E+01**		**+**
FocA	CU_fimbriae	9,29E+02		+
**FocB**	**CU_fimbriae**	**1,97E+01**		**+**
FocC	CU_fimbriae	1,07E+04		+
FocD	CU_fimbriae	1,07E+04		+
FocF	CU_fimbriae	1,07E+04		+
FocG	CU_fimbriae	4,29E+03		+
FocH	CU_fimbriae	4,44E+04		+
FocI	CU_fimbriae	3,06E+04		+
FocX	CU_fimbriae	9,42E+03		+
FocY	CU_fimbriae	9,42E+03		+
HtrE	CU_fimbriae	3,77E+04		+
LpfB1	CU_fimbriae	4,46E+04		+
LpfD1	CU_fimbriae	2,71E+04		+
PapA	CU_fimbriae	4,07E+04		+
PapD	CU_fimbriae	1,82E+04		+
PapE	CU_fimbriae	1,82E+04		+
PapF	CU_fimbriae	1,52E+04		+
PapGI	CU_fimbriae	1,52E+04		+
PapI	CU_fimbriae	9,29E+02		+
PapJ	CU_fimbriae	2,82E+04		+
PapK	CU_fimbriae	1,82E+04		+
PapX	CU_fimbriae	3,03E+03		+
SfaA	CU_fimbriae	9,29E+02		+
**SfaB**	**CU_fimbriae**	**1,97E+01**		**+**
SfaE	CU_fimbriae	1,07E+04		+
SfaF	CU_fimbriae	1,07E+04		+
SfaG	CU_fimbriae	1,07E+04		+
SfaS	CU_fimbriae	4,29E+03		+
YadN	CU_fimbriae	1,49E+04		+
YgiL	CU_fimbriae	3,03E+03		+
CjrB	Iron_uptake	3,52E+03		+
CjrC	Iron_uptake	3,38E+03		+
FhuA	Iron_uptake	2,79E+04		+
EtsA	Transport	2,26E+03	+	
EtsB	Transport	3,20E+03	+	
EtsC	Transport	3,13E+03	+	
CofR	Type-4-pilus	7,79E+03	+	
Etp	Groupe 4 capsule	4,02E+04	+	
GfcA	Groupe 4 capsule	2,70E+04	+	
GfcB	Groupe 4 capsule	2,70E+04	+	

GfcC	Groupe 4 capsule	2,70E+04	+	
GfcD	Groupe 4 capsule	2,70E+04	+	
GfcE	Groupe 4 capsule	2,70E+04	+	
KpsC-III	Group II capsule	1,03E+04	+	
KpsC-II	Group II capsule	2,70E+04		+
KpsD-II	Group II capsule	2,70E+04		+
KpsE-II	Group II capsule	2,70E+04		+
KpsM-II-K5	Group II capsule	2,75E+04		+
KpsM-K1	Group II capsule	4,31E+04		+
KpsM-K2	Group II capsule	4,03E+04		+
KpsS-II	Group II capsule	2,70E+04		+
KpsT-II-K5	Group II capsule	1,03E+04		+
KpsU-II	Group II capsule	2,70E+04		+
NeuS	Group II capsule	9,42E+03		+
GspB	T2SS	2,83E+03		+
GspL	T2SS	2,22E+04		+
GspM	T2SS	2,22E+04		+
EspF	T3SS	1,90E+04		+
EspL2	T3SS	7,12E+03	+	
EspX5	T3SS	3,21E+04	+	
Cah	T5SS	7,89E+03	+	
Flu_2	T5SS	1,66E+04	+	
Saa	T5SS	2,79E+04	+	
Tsh	T5SS	5,30E+03		+
UpaB	T5SS	2,22E+04		+
Aec14	T6SS	4,44E+04		+
Aec15	T6SS	2,98E+04		+
APECO1_1753	T6SS	2,98E+04		+
ClbA	Toxins	6,04E+03		+
ClbB	Toxins	6,04E+03		+
ClbC	Toxins	6,04E+03		+
ClbD	Toxins	6,04E+03		+
ClbE	Toxins	6,04E+03		+
ClbF	Toxins	6,04E+03		+
ClbG	Toxins	6,04E+03		+
ClbH	Toxins	6,04E+03		+
ClbI	Toxins	9,42E+03		+
ClbJ	Toxins	4,29E+03		+
ClbK	Toxins	6,04E+03		+
ClbL	Toxins	6,04E+03		+
ClbM	Toxins	6,04E+03		+
ClbN	Toxins	6,04E+03		+
ClbO	Toxins	6,04E+03		+
ClbP	Toxins	6,04E+03		+
ClbR	Toxins	6,04E+03		+
Cnf3	Toxins	6,04E+03		+
EltB	Toxins	3,38E+03		+
SenB	Toxins	3,38E+03		+

VFs indicated in bold remained significantly associated with the control group even after Bonferroni correction.

Supplementary Table 4. Resistance genes (RGs) with the largest differences between KTx and control isolates.

RG	Group	-		+		P
		No. of isolates	%	No. of isolates	%	
blaTEM1B	Controls	85	76.6	26	23.4	**0.0003**
	KTx	36	50.7	35	49.3	
strA	Controls	92	82.9	19	17.1	**0.0097**
	KTx	47	66.2	24	33.8	
strB	Controls	92	82.9	19	17.1	**0.0015**
	KTx	44	62.0	27	38.0	
sul2	Controls	85	76.6	26	23.4	0.0841
	KTx	46	64.8	25	35.2	

No.= number, KTx= strains of kidney transplanted patients, p values were obtained using Fisher's exact test.

Supplementary Table 5. Distribution of the rdar/ras, saw and mucoid morphotypes expressed by KTx and control UPEC isolates.

		**rdar/ras**		**saw**		**mucoid**	
		**No. of isolates**	%	No. of isolates	%	No. of isolates	%
RT	KTx	47	66.2	6	8.5	8	11.3
	Controls	68	61.3	19	17.1	7	6.3
	p	0.5007		0.0976		0.2352	
30°C	KTx	41	57.7	8	11.3	9	12.7
	Controls	57	51.4	29	26.1	7	6.3
	p	0.3986		**0.0151**		0.1388	
37°C	KTx	29	40.8	17	23.9	11	15.5
	Controls	41	36.9	38	34.2	7	6.3
	p	0.5971		0.1403		**0.0429**	

No.= number, KTx= strains of kidney transplanted patients, RT= room temperature, p values were obtained using Fisher's exact test.

Supplementary Table 6. Calcofluor binding KTx and control UPEC isolates.

		CF-positive	
		No. of isolates	%
RT	KTx	61	85.9
	Controls	79	71.2
	p	**0.0213**	
30°C	KTx	53	74.6
	Controls	72	64.9
	p	0.1651	
37°C	KTx	45	63.4
	Controls	63	56.8
	p	0.3749	

CF=Calcofluor, No.= number, KTx= strains of kidney transplanted patients, p values were obtained using Fisher's exact test.

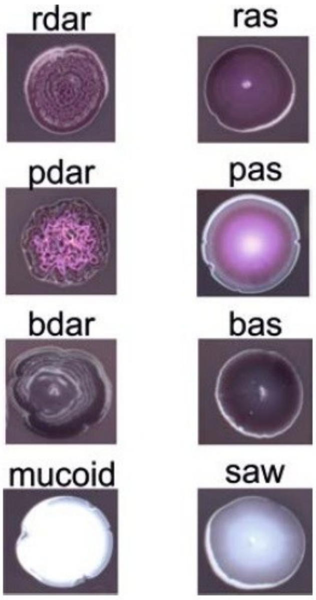

	cellulose	curli
rdar = red, dry and rough	+	+
ras = red and smooth	+	(+)
pdar = pink, dry and rough	+	-
pas = pink and smooth	(+)	-
bdar = brown, dry and rough	-	+
bas = brown and smooth	-	(+)
mucoid	+/-	+/-
saw = smooth and white	-	-

Supplementary Fig. 1. Representative biofilm morphotypes expressed by UPEC isolates on Congo Red agar plates.

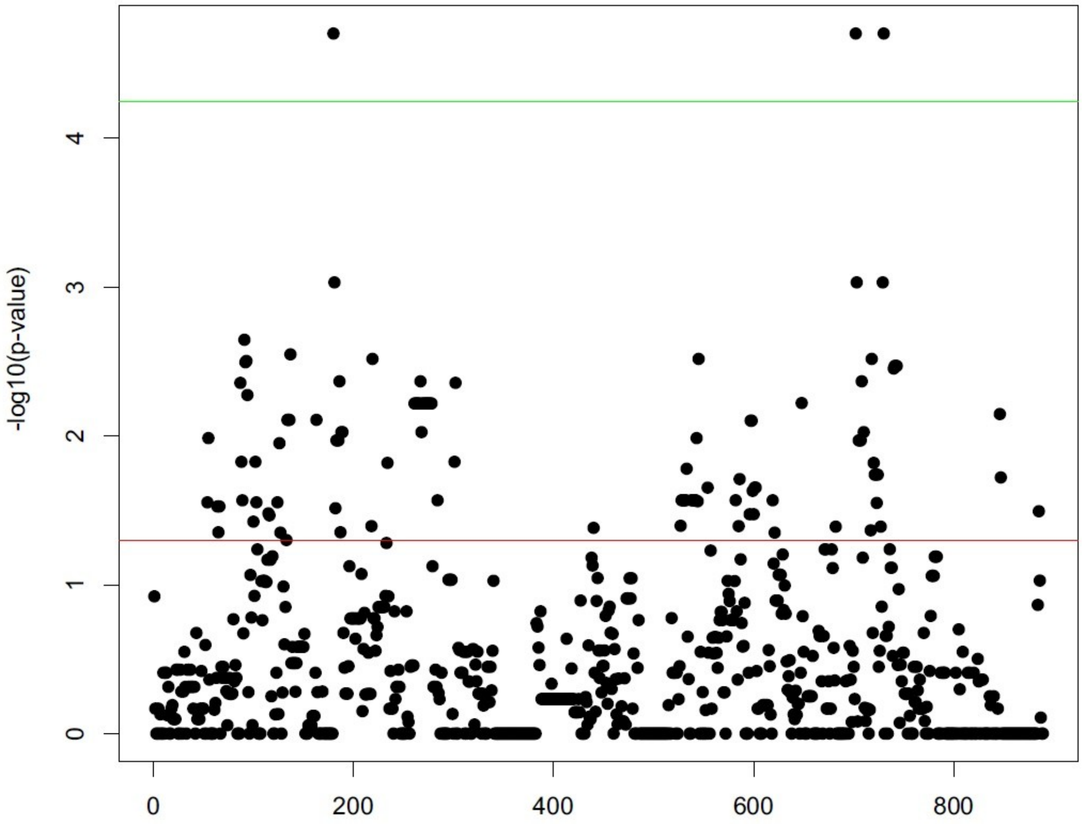

Supplementary Fig. 2. Manhattan Plot of Fisher's exact test *p*-values for the prevalence of individual virulence factors (VF) in an isolate group (KTx or control). The red line separates VFs with *p*-values <0.05 from VFs without significant association with an isolate group. The green line separates VFs with significant association ($p<0.05$) after the Bonferroni correction.

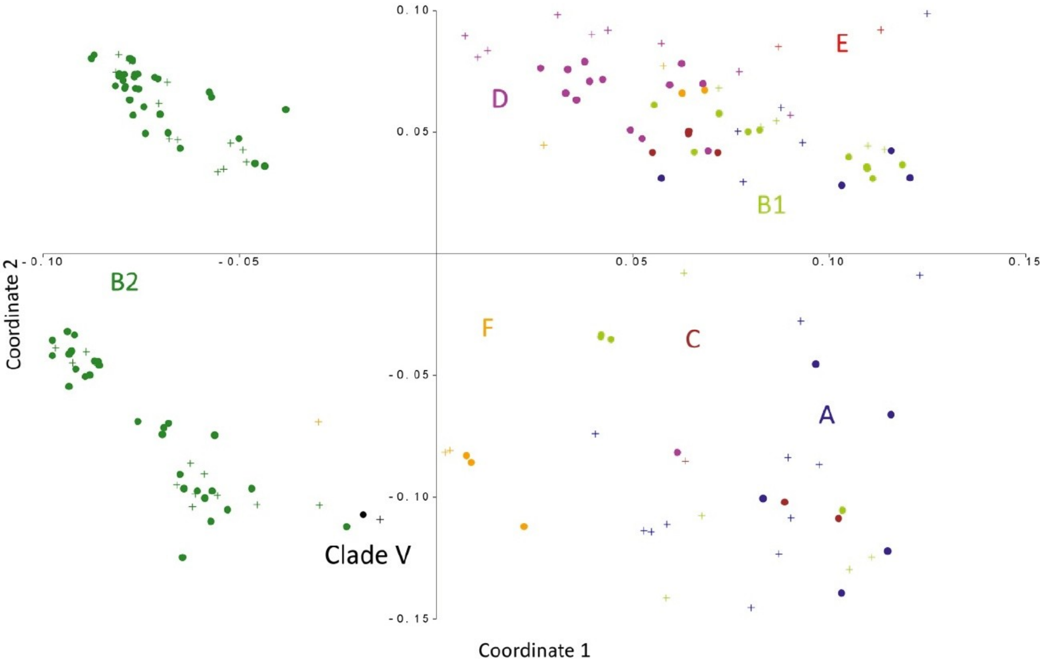

Supplementary Fig. 3. Principal Coordinates Analysis (PCoA) to examine the grouping of UPEC isolates according to the presence/absence of virulence-associated genes, their phylogenetic group and their isolate group (KTx vs. control group). The axes are scaled with eigenvalue scaling using the

square root of the eigenvalue and indicate the percentage of variation explained in the PCoA. KTx strains are marked with (+) and control strains are marked with (•). The phylogroup of each strain is color coded (phylogroup A = blue, phylogroup B1 = light green, phylogroup B2 = dark green, phylogroup C = brown, phylogroup D = pink, phylogroup E = red, phylogroup F = orange, clade V = black).
